# Comparative Experiments of V2X Security Protocol Based on Hash Chain Cryptography

**DOI:** 10.3390/s20195719

**Published:** 2020-10-08

**Authors:** Shimaa A. Abdel Hakeem, Mohamed A. Abd El-Gawad, HyungWon Kim

**Affiliations:** 1 School of Electronics Engineering, Chungbuk National University, Cheongju 28644, Korea; shimaakotb@cbnu.ac.kr; 2 Electronics Research Institute (ERI), Giza 12622, Egypt; 3 School of Electrical Engineering, Korea University, Seoul 02841, Korea; mgawad@korea.ac.kr; 4 National Telecommunication Institute, Cairo 11768, Egypt

**Keywords:** MAC algorithm, hash-chain, privacy, commercial V2X devices, authentication, IEEE1609.2, ETSI-103-097

## Abstract

Vehicle-to-everything (V2X) is the communication technology designed to support road safety for drivers and autonomous driving. The light-weight security solution is crucial to meet the real-time needs of on-board V2X applications. However, most of the recently proposed V2X security protocols—based on the Elliptic Curve Digital Signature Algorithm (ECDSA)—are not efficient enough to support fast processing and reduce the communication overhead between vehicles. ECDSA provides a high-security level at the cost of excessive communication and computation overhead, which motivates us to propose a light-weight message authentication and privacy preservation protocol for V2X communications. The proposed protocol achieves highly secure message authentication at a substantially lower cost by introducing a hash chain of secret keys for a Message Authentication Code (MAC). We implemented the proposed protocol using commercial V2X devices to prove its performance advantages over the standard and non-standard protocols. We constructed real V2X networks using commercial V2X devices that run our implemented protocol. Our extensive experiments with real networks demonstrate that the proposed protocol reduces the communication overhead by 6 times and computation overhead by more than 100 times compared with the IEEE1609.2 standard. Moreover, the proposed protocol reduces the communication overhead by 4 times and the computation overhead by up to 100 times compared with a non-standard security protocol, TESLA. The proposed protocol substantially reduces the average end-to-end delay to 2.5 ms, which is a 24- and 28-fold reduction, respectively, compared with the IEEE1609 and TESLA protocols.

## 1. Introduction

Recently, vehicle-to-everything (V2X) communication technologies have been developed to enhance road safety by exchanging messages about vehicle mobility and road status. The messages are continuously broadcasted among many vehicles under high mobility conditions, which makes the V2X network susceptible to security attacks. V2X network, therefore, needs a robust security framework to allow vehicles to communicate securely and reliably. Many security protocols recently published are aimed at enhancing the security level of the V2X network by satisfying the challenges and requirements of existing V2X security standards. This paper is concerned with V2X authentication and privacy preservation, which are among the most critical V2X security problems. V2X authentication is a critical requirement to manage authorizing the access to V2X network resources. It allows only authenticated vehicles to exchange road statues messages securely [1].

Many V2X security protocols are based on public key infrastructure (PKI) employing elliptic curve cryptography (ECC) to support message authentication and integrity [2]. In PKI, the elliptic curve digital signature algorithm (ECDSA) generates a signature for the message to send and attaches the sender’s certificate along with the generated signature to the original message. While the security protocols based on ECDSA provide a high-security level, they incur high communication cost due to the large size of the attached certificate and digital signature [3]. The signing and verifying processes of the ECC method are also highly time-consuming, especially when implemented in software. One of the critical issues concerning the previous V2X authentication protocols is their heavy dependence on a centralized certificate authority (CA), which can cause severe traffic congestion and a single point of failure at the CA.

On the other hand, some V2X security protocols are proposed to decentralize the CA tasks and reduce the communication and computation overhead of PKI-based solutions. Recently, some security protocols suggest using message authentication code (MAC) instead of the ECDSA to reduce the security overhead by attaching a short hashed-signature to each message [4,5]. Conventional MAC algorithms calculate the MAC digest over the plain text message using a secret key shared between the sender and receiver. The receiver side calculates the MAC digest over the received message using the same shared key and compares the calculated digest with the received one to accept or reject the message [6]. Although the conventional MAC algorithm is a light-weight solution with fast hash calculation, it is highly vulnerable to shared-key sniffing attacks. According to the IEEE1609.2 standard, each on-board unit (OBU) is equipped with a tamper proof device (TPD) or hardware security module (HSM), to securely keep the cryptographic material and private keys [7]. TPDs are generally assumed impossible to be compromised and thus becoming an important component in each vehicle. Other proposals also suggested using biometric devices (BDs) to provide security services for vehicles and reduce the communication overhead on the CA [8].

In [9], we proposed a lightweight V2X security protocol-based simulation and utilized the BD and TPD devices to generate dynamic pseudo-identities and random hashed keys. The analysis of our proposed security protocol [9] showed that it supports integrity, privacy, and authentication using a lightweight MAC signature per message and a pregenerated hash table. The proposed protocol enables vehicles to store the same hash chain table of *n* hashed signing elements. Each sender randomly chooses one key from the hash chain table to sign the message, and it attaches only the key index to the message as a pointer to the key in the hash table. The results of the software implementation based on NS-3 showed that the proposed protocol significantly improves the computation speed, and reduces the network overhead. The proposed protocol reduced the communication overhead by 20–85% compared to other protocols proposed in [10,11,12,13]. The results also showed that the proposed protocol could sign 60,000 messages per second, which is 55 times faster than the previous protocols.

For this paper, to prove the performance advantage of our proposed protocol [9], we implemented it using commercial V2X OBU and RSU devices and conducted V2X network tests with the implemented protocol. We measured the performance of the ETSI-103-097 and IEEE1609.2 standards, which employ the ECDSA algorithm to sign and verify V2X messages. We then compared the proposed work [9] and the standard protocols in terms of the communication and computation cost measured from the commercial V2X devices. To the best of our knowledge, no previous paper has reported experimental results of V2X security protocols using a real network with commercial V2X devices.

In summary, the contributions of this paper are as follows:

- Implementing our light-weight hash-based V2X authentication protocol on commercial V2X devices.
- Analyzing the performance of existing V2X security standards of IEEE1609.2 and ETSI-103-097 using commercial V2X devices.
- Comparing the communication cost in terms of message size for the proposed protocol and standard security protocols using commercial V2X devices.
- Comparing the performance between the proposed protocol and the standard protocol in terms of end-to-end delay and packet verification ratio.
- Comparing the communication and computation cost of the proposed protocol and non-standard security protocols using commercial V2X devices.

The rest of this paper is organized as follows: Section 2 describes the previous V2X security protocols and security standards. Section 3 presents the proposed protocol architecture and the proposed authentication. Section 4 describes the proposed message structure and communication overhead. In Section 5, the test platform is given, whereas computation overhead is analyzed in Section 6. Communication overhead performance is assessed in Section 7, while the communication and computation overhead of non-standard security protocols are given in Section 8. Conclusions are provided in Section 9.

## 2. Related Work

In this section, we present recent non-standard and standard security protocols that are based on the message authentication code (MAC)- and the elliptic curve digital signature algorithm (ECDSA)-based protocols.

### 2.1. Non-Standard V2x Security Protocols

In this section, we describe the previous security protocols that incorporate Tamper proof devices (TPDs) to decentralize the CA tasks of signing and verifying messages. Although many recently V2X security methods have addressed the decentralization of the CA tasks, most of the works report their methods and performance only via either simulations or mathematical analysis [9,10,11,12,13].

In [10], the authors proposed a conditional privacy-preserving authentication (CPPA) protocol based on bilinear pairing cryptography to improve the identity-based security in V2X. However, this protocol cannot prevent message replay and modification attacks. In [11], the authors assumed a single secret system key and kept it securely in the TPD device to prevent key sniffing attacks.

Using a single system key is considered to be vulnerable to a point of failure for the system. In [12], Wang et al. proposed a 2FLIP protocol to provide a lightweight authentication and privacy solution. In the case of a multi-driver revocation process, 2FLIP cannot recognize which driver misbehaves and revokes the whole vehicle regardless of the drivers, which results in an inaccurate revocation process.

In [13], the authors proposed an efficient authentication and privacy protocol based on the key-separation algorithm. Their protocol provides two types of secret keys and updates them periodically. The first secret key is issued by the CA of the network system, while the second secret key is issued by each vehicle. This protocol supports TPD installation in each vehicle; however, it is vulnerable to single system key failure and modification attacks.

In [14], the authors proposed a secure multicast authentication protocol called TESLA. TESLA is based on symmetric cryptography (e.g., message authentication code algorithm with delayed key disclosure). The TESLA protocol satisfies low communication overhead requirements, however, it cannot support the non-repudiation function and incurs high delay due to the delayed disclosure of shared keys.

In [15], the authors proposed a security protocol called VAST that provides multi-hop authentication for V2X applications. VAST is based on a mix of TESLA protocol and the ECDSA algorithm.

In [16], Huang et al. proposed the anonymous batch authenticated and key agreement (ABAKA) protocol to support simultaneous multi-request authentication using individual session keys for each vehicle based on ECDSA.

In [17], Biswas et al. proposed an ID-based authentication protocol using proxy signatures for V2X safety applications. The protocol of [17] uses vehicle location information as a signer’s ID to sign and verify the messages. It supports only single-zone communication which can cause a critical problem.

In [18], Tzeng et al. proposed an improved ID-based privacy-preserving and authentication protocol for V2X. It resists to some security attacks and proposed a secure solution using the random oracle model.

In [19], the lightweight efficient strong privacy-preserving protocol (LESPP) was proposed for securing V2X communication using symmetric encryption and MAC algorithm. LESPP provides a light-weight authentication protocol; however, it can’t resist the single system key failure problem.

### 2.2. Standard V2X Security Protocols

In this section, we introduce the security standards implementation in ETSI-103-097 and IEEE1609.2. Both standards attach an ECDSA signature and a digital certificate in every transmitted message to provide message and identity authentication. Security protocols based on digital signatures impose high communication overhead and high computation cost. Several related works have benchmarked the signing and verifying procedures of the ECDSA protocol proposed by IEEE1609.2 and ETSI-103-097 on different processors [20,21,22,23]. However, few works reported the field performance of V2X standard protocols on real V2X wireless devices, which motivated us to measure the field performance of the standard in a real V2X testbed. In [24], the PRESERVE V2X project implemented the ECDSA algorithm on FPGA to evaluate the signature and verification time. The results showed that the ECDSA could generate 400 signatures per second and perform 30 verifications per second. Similarly, the authors in [25] implemented the ETSI-103-097 standard and analyzed different complexities in the standard.

In [26], the VSC-A project evaluated the IEEE 1609.2 standard in a testbed implemented on a PC platform and showed that the signature generation time and verification time based on ECC-256 is 6.6 ms and 28.5 ms, respectively. Their experimental analysis indicates that the IEEE 1609.2 standard based on ECDSA is highly demanding in the resource. In [27], the authors reported software implementation of the IEEE 1609.2 standard using the OpenSSL security library.

To the best of our knowledge, no publication reports the real implementation of the V2X security protocols based on commercial V2X wireless devices. Thus, we implemented and tested a light-weight authentication protocol that supports messages authentication and integrity using commercial V2X wireless devices. We also analyzed the ETSI-103-097 and IEEE1609.2 security standards using commercial V2X wireless devices and compare their performance with the proposed protocol performance.

## 3. Proposed Protocol: Architecture and Algorithm

In this section, we describe the proposed protocol’s basics and authentication algorithm.

### 3.1. The Architecture of the Proposed Protocol

In [9], we proposed a decentralized lightweight authentication protocol for vehicle-to-vehicle (V2V) communications. The proposed protocol can preserve privacy by introducing a self-generation of pseudo-identities instead of large digital certificates. Moreover, we assume that each vehicle is equipped with BD and TPD security devices with a pre-stored shared hash table of signing keys. The use of security devices plays the role of CA agents and can generate pseudo-identities with corresponding private keys. Pseudo-identities are used to protect the driver’s privacy and hide real identity to avoid being tracked. The use of a pre-stored shared hash table provides an efficient message integrity solution that calculates a light-weight MAC signature over each message using the stored hashed keys without disclosing the keys for the receivers. The proposed protocol satisfies the most critical V2X security requirements, such as integrity, authentication and conditional traceability. Moreover, it provides a revocation solution to report the misbehaving vehicles to the CA, which can determine the real identity of the accused vehicle from a pre-stored vehicle database. The revocation method in this protocol supports the multi-driver revocation scenario and only revokes the accused driver’s pseudo-identity instead of canceling the vehicle identity. In [9], we implemented the proposed protocol using the NS-3 simulator and Miracle crypto library to evaluate the target security functions.

**Figure 1:**
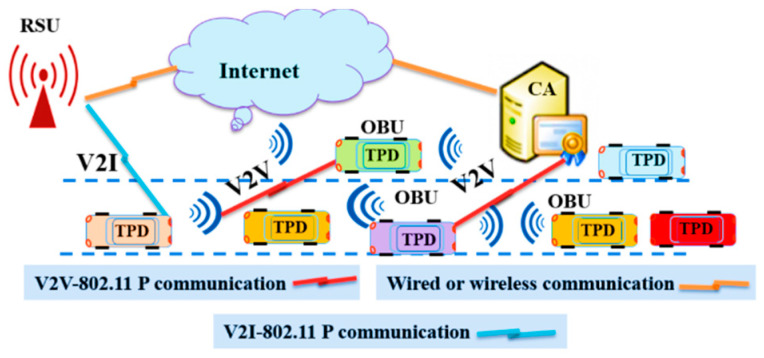
V2V and V2I communication modes in the proposed protocol.

In Figure 1, we describe the proposed protocol’s system model that supports two vehicular communication modes, vehicle-to-vehicle (V2V) and vehicle-to-infrastructure (V2I). The proposed protocol supports V2I mode when a road side unit (RSU) is present in the communication range of vehicles to offer road safety services. The system model consists of a CA, a set of RSUs distributed on the roads, and some vehicles on the road. The role of CA includes the initialization, authentication, system key updating process, and vehicle revocation process. To support a car-sharing service with multiple driver scenarios effectively, each driver is required to register at the CA individually through a secure channel. Then the CA approves the driver registration and configures the BD device.

**Figure 2:**
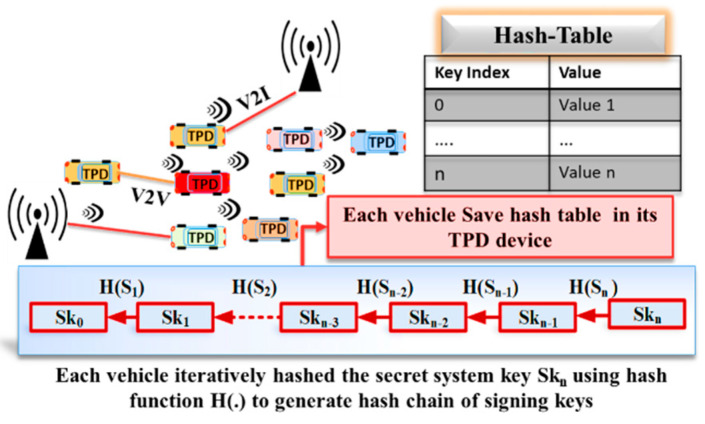
The generated hash chain keys.

**Figure 3:**
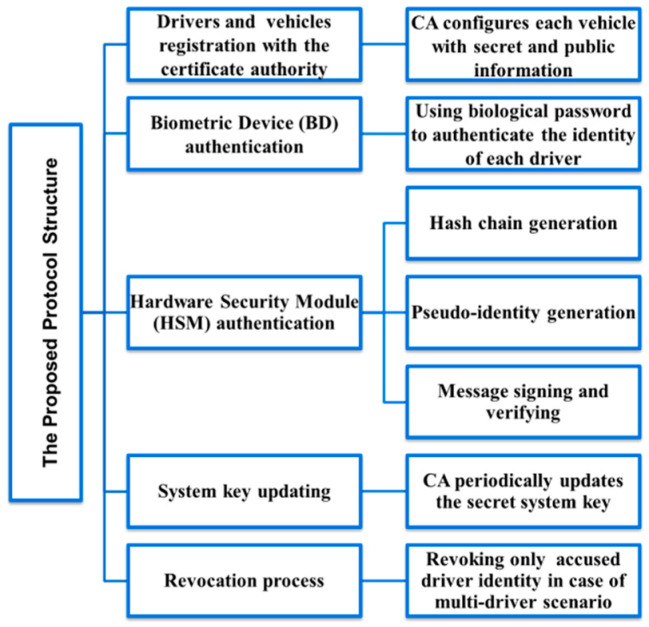
The proposed protocol full architecture.

In the initialization phase of the proposed protocol, each vehicle generates a hash chain using an initial secret key provided by the CA at the registration phase. Using a pre-defined hash function, the TPD device in each vehicle iteratively calculates the hashes for corresponding secret keys $sk_{n}$. It securely generates a hash chain of *n* elements to determine the signing hashed keys. The shared system key is pre-stored during the registration phase using a hash function H (.), as shown in Figure 2. The overview of the proposed protocol is illustrated in Figure 3. For further details, refer to [9].

We summarize the advantages of the proposed protocol as follows:

- It preserves privacy by generating a dynamic pseudo-identity (PID) to hide the real identity of the vehicles.
- It securely hides the secret keys by utilizing a pre-generated hash-chain, in contrast to the conventional HMAC, which discloses the keys.
- It provides lower communication overhead with a significantly shorter message than standard messages.
- It supports both authentication and privacy with substantially lower computation overhead than standards by effectively integrating PID, hash-indexed key, and HMAC, so it eliminates the needs for expensive signature encryption.

### 3.2. Algorithm of the Proposed Authentication

The proposed algorithm employs a light-weight authentication method called Keyed Hash Message Authentication Code (HMAC) for message authentication and integrity. We implemented the HMAC-SHA256 authentication protocol to sign and verify messages at the sender and the receiver. Our authentication algorithm is illustrated in Algorithm 1. The parameters used in Algorithm 1 are described below:

- $HMAC\_SHA256{\;}_{k_{i}}$: MAC tag value calculated over message m using a signing key $k_{i}$.
- $Sig_{ki}$: Output signature of the MAC operation that is truncated to only 12 bytes.
- $m_{i,j}$: Message to be signed and transmitted from the vehicle $v_{i}$ to a vehicle $v_{j}$.
- $PID_{i}$: The Pseudo-identity of the sender vehicle $v_{i}$.
- $T_{s}$: Current timestamp.

**Algorithm 1** Proposed Message Authentication Algorithm**Requirements:** two vehicles $v_{i}$, $v_{j}$ share a common hash-chain of n secret keys **Signing Algorithm:** $v_{i}$ pick a random secret key $k_{i}$ from the pre-stored hash table. $v_{i}$ calculates a MAC signature $\left(Sig_{k_{i}}\right)$ using HMAC-SHA256 with the key $k_{i}$ over the message consisting of pseudo-identity $PID_{i}$, the original payload $m_{i,j}$, and time-stamp $T_{s}$, $Sig_{k_{i}}=HMAC\_SHA256{\;}_{k_{i}}\;(PID_{i}\left|\left|m_{i,j}\right|\right|Ts$) $v_{i}$ sends {$PID_{i},\;Sig_{k_{i}},\;m_{i,j},k_{index},\;T_{s}$} to $v_{j}$. **Verifying Algorithm:**
1.$v_{j}$ receives {$PID_{i},\;Sig_{ki},\;m_{i,j},k_{index},\;T_{s}$}, then extract the $k_{index}$, search the pre-stored hash table to find $\;k_{i}$ that match the received index.2.$v_{j}$ calculates a MAC signature
$Sig_{k_{i}}^{*}=HMAC\_SHA256{\;}_{k_{i}}\;\left(PID_{i}\left|\left|m_{i,j}\right|\right|Ts\right)$.
3.  $v_{j}$ accepts the message if and only if $Sig_{k_{i}}=Sig_{k_{i}}^{*}$, else discard the message.


**Figure 4:**
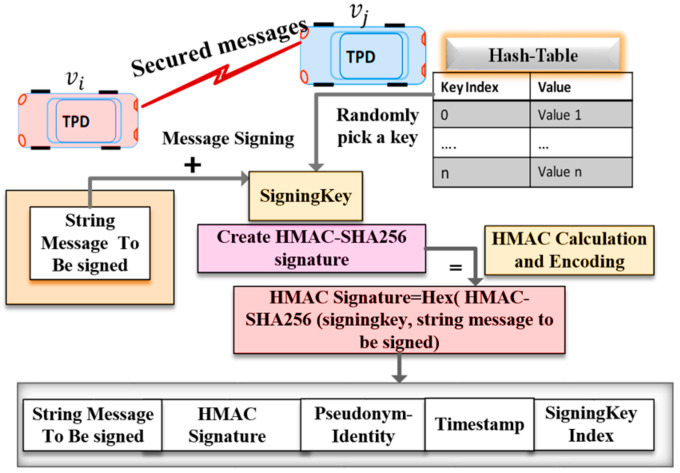
Message Signing Procedure.

**Figure 5:**
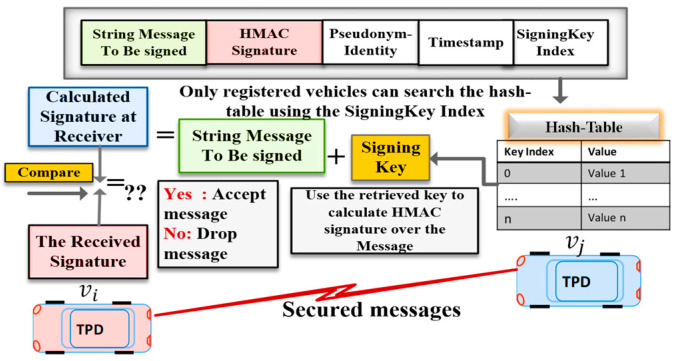
Message Verification Procedure.

As the sender vehicle $v_{i}$ calculates the signature $Sig_{ki}$ over the message using a random key $k_{i}$ retrieved from the hash table. Then $v_{i}$ attaches the calculated signature, the signing key index, $k_{index}$, the current timestamp, $T_{s}$, and the sender pseudo-identity, and $PID_{i}$, to each transmitted message, as shown in Figure 4. When the receiver vehicle $v_{j}$ receives the signed message {$PID_{i},\;Sig_{k_{i}},\;m_{i,j},\;k_{index},\;T_{s}$}, $v_{j}$ checks the freshness of timestamp Ts. If $T_{s}$ is invalid, $v_{j}$ rejects the message; otherwise, $v_{j}$ verifies the signature of the received message, as shown in Figure 5.

We implemented our proposed protocol by modifying the IEEE 1609 protocol stack and utilizing the network layer and MAC layer of the SDK of the V2X device. The key difference of our protocol from the standard security protocol lies in our light-weight algorithm for message authentication and integrity checking based on the HMAC-SHA256. In contrast, standard security protocols employ the computation-intensive ECDSA algorithm to provide authentication and non-repudiation security requirements. The fast authentication process of the proposed method is also attributed to our efficient authentication key generation method. Security algorithms can provide different security strength depending on the combination of the algorithms and the key size used.

The security strength of security protocols is often defined by the number of bits, as *n* bit security indicates that an attacker must perform 2*^n^* trials to break this protocol. The proposed protocol can provide a security strength up to 256-bits by using a combination of the HMAC-SHA256 algorithm and a key-size of 256-bits [28,29]. On the other hand, the standard protocols based on a combination of the ECDSA algorithm and an ECC key-size of 256 support a security strength of 128-bits according to the National Institute of Standards and Technology (NIST) [30]. Using the fast and secure algorithm described above, the proposed protocol can ensure message authentication with high-security strength.

## 4. Analysis of Communication Overhead

As described above, V2X security standards employ an ECDSA certificate and signature to provide identity and message authentication. On the other hand, the proposed protocol utilizes a pseudo-identity to support identity authentication and HMAC signature to provide message authentication. In the following subsections, we analyze the communication overhead of the three protocols: the proposed protocol, IEEE 1609.2, and ETSI-103-097.

### 4.1. The Proposed Protocol

In this section, we present the communication cost analysis of the proposed protocol by calculating the transmitted message size. For the proposed protocol, the authentication header of V2V transmitted messages is represented by Equation (1):

(1) $$PID_{i}\left|\left|Sig_{ki}\right|\right|Key_{index}||T_{s}$$

here, $PID_{i}$ indicates a sender $v_{i}$’s pseudo-identity with a condition $PID_{i}$ *∈* finite field (Z^*^_q_). The use of pseudo ID hides the real identity and preserves the privacy of $v_{i}$, and thus allows vehicles to communicate without being tracked. In the proposed protocol, the pseudo-identities and signing keys are pre-generated offline and pre-stored in OBUs. Therefore, the computation time for generating pseudo-identifies and keys does not incur any overhead in OBUs during the V2X communication. Pseudo-identities are short size random numbers of 20 bytes to match the use of certificates in standards that provides identity authentication. The random number generation is substantially lower cost than the computationally expensive short-lived certificates used by the current standards. $Sig_{ki}$ represents the truncated HMAC signature of 12 bytes calculated over the message. As the security strength supported by the HMAC signature depends on its length, the length of the signature must be sufficiently long to prevent the acceptance of malicious messages. For most applications, a length of 64 to 96 bits is acceptable and sufficient. As recommended by NIST, we select only the leftmost 12 bytes of the 32 bytes HMAC hash size while discarding the 20 bytes, which causes very little sacrifice of the HMAC’s security level [31].

$Key_{index}$ represents the index to the 4-byte hashed key in the hash table, while *T_S_* represents a timestamp of 4 bytes to prevent the retransmission of old messages like replay attacks. The signing key size in our implementation is 256-bit since it employs an SHA-256 hashing function. Each registered vehicle has the pre-stored hash-table, which can retrieve the signing key from a given index. Therefore, in the proposed method, the sender only transmits the index of the signing key. Then, the receiver retrieves the key from the pre-stored hash-table using the received index, and it verifies the integrity of the message.

**Figure 6:**
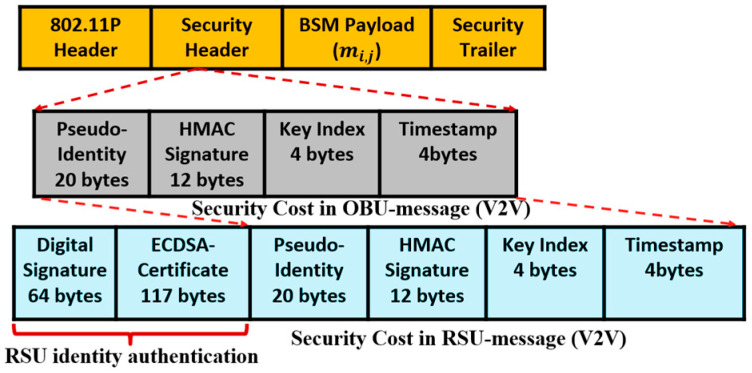
The proposed protocol’s message format in the case of OBU and RSU.

Thus, the overhead of message size for the V2V authentication is 20 (pseudo ID) + 12 (HMAC signature) + 4 (key index) + 4 (timestamp) = 40 bytes. Our regular V2V messages employ a short size pseudo-identity to authenticate the hide the real identity and avoid tracking. Although our protocol aims at minimizing the use of ECDSA protocol, some rare events—system key update and vehicle revocation—require vehicle-to-infrastructure (V2I) messages that employ ECDSA certificate to authenticate the RSU. At the beginning of communication, an RSU authenticates itself by broadcasting an ECDSA certificate of 117 bytes and ECDSA-256 signature of 64 bytes, as described in the security protocol standards [22], so the total security overhead for V2I messages is 64 (ECDSA signature) + 117 (ECDSA certificate) + 12 (HMAC signature) + 4 (key index) + 4 (timestamp) = 201 bytes. Figure 6 shows the V2V message format and the V2I message format of the proposed protocol.

### 4.2. IEEE 1609.2 Standard

In the IEEE1609 V2X standard, OBU devices transmit various SAE-J2735 beaconing messages [32]. The most common message type in V2V scenarios is a basic safety message (BSM) that broadcasts the vehicle information, including the position, speed, and heading.

Each OBU sends BSM messages at a rate of 10 Hz or with an interval of 100 ms. A BSM with a full certificate is transmitted once in every 5 transmissions (which corresponds to approximately every 500 ms), while the other BSMs are sent with only a certificate digest to reduce the overhead. In the IEEE1609 experiments, we observed that the security overhead for BSM messages ranges between (93–180) bytes. Carrying only certificate digest incurs a security overhead of 90 bytes while carrying a full certificate incurs a higher overhead of 180 bytes.

Concerning RSU message types such as wave service advertisements (WSAs), and signal phase and timing message (SPAT), we analyzed the security overhead of these messages by enabling the secure broadcast in the RSU device.

**Figure 7:**
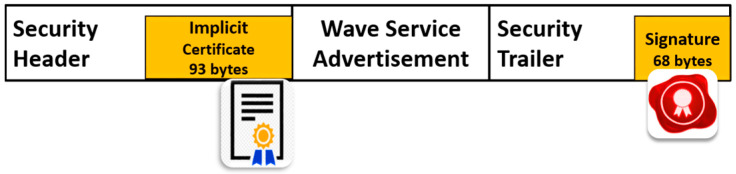
WSA messages signed with implicit certificate in IEEE1609.2.

For WSAs, the RSU broadcasts one message every 100 ms where an ECDSA implicit certificate is attached, as shown in Figure 7. The size of WSA security header fields (e.g., implicit certificate) and security trailer fields (signature) in our experiments is 232 bytes. We have also measured the overhead of signal phase and timing message (SPAT), yet another type of SAE-J2735 standard messages broadcasted by RSU at a rate of 100 ms. For the signed SPAT attached with a full certificate, the security overhead grows to 224 bytes.

### 4.3. ETSI-103-097 Standard

In the ETSI V2X standard, OBU devices transmit cooperative awareness message (CAMs) that broadcast the vehicle information, including speed, position, and heading. In the case of an RSU, it sends decentralized environmental notification messages (DENMs) (e.g., road work warnings or adverse weather warnings), MAP/signal phase and timing (MAP/SPAT) messages from traffic lights, and in-vehicle information (IVI). In the ETSI V2X standard, the secured CAM message consists of a collection of data elements that are mandatory information, optional container, signature to provide message integrity, and a certificate to support identity authentication.

**Figure 8:**
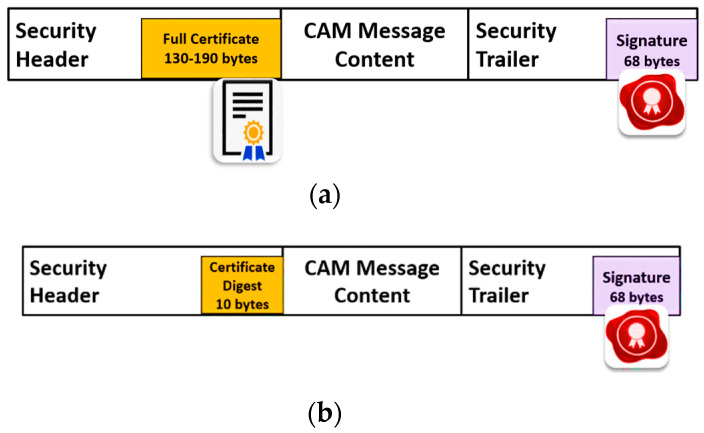
(**a**) Secure CAM message with authorization ticket; (**b**) Secure CAM message with certificate digest using SHA-256.

In Figure 8a, a secure CAM packet with a full certificate is presented, and Figure 8b shows the CAM message with a certificate digest. Upon receiving the full certificate in the first CAM packet, each OBU keeps the digest value of the received certificate’s hash to reduce the future verification time and message overhead of the full certificate in the foregoing communication. The majority of CAM message carries only the digest of the certificate’s hash to avoid the overhead of carrying the entire certificate. Upon receiving the future CAM packet, the receiver searches the stored digests of certificates to match the corresponding received certificate and verify the sender. In the ETSI-103-097 security standard, the full certificate size ranges between (130–190) bytes, while the certificate digest size is only 8 bytes. Carrying only the certificate digest reduces the security overhead of CAM’s message authentication. In our experiments, the security overhead represents the size of security header fields (e.g., the certificate or certificate digest, certificate information, and issuer information), and security trailer fields (e.g., the signature). We observed that the security overhead ranges between (90–234) bytes. Carrying only the certificate’s digest introduces a security overhead of only 90 bytes while carrying a full certificate introduces a higher overhead of 234 bytes. Although the use of certificate digest can reduce the overhead to 90 bytes, it is still significantly higher than 40 bytes overhead of the proposed protocol.

In ETSI-103-097 Standard, RSU devices broadcast additional message types such as DENM, SPAT, MAP, and IVI message types. The security overhead consists of a security header and a security trailer. In the case of DENM messages, the security header size is 199 bytes, and the security trailer size is 68 bytes, which leads to a total overhead of 267 bytes. In the case of the SPAT messages, the security header size is 198 bytes, and the security trailer size is 68 bytes producing a total overhead of 266. In the case of IVI and MAP messages, the security header size is 166 bytes, and the security trailer size is 68 bytes resulting in a total overhead of 234 bytes.

Table 1 summarizes the communication overhead for the proposed protocol and standards. From the above overhead measurement and analysis, we found that the proposed protocol incurs an overhead of only 40 bytes for OBU messages, which is up to 6 times reduction compared with standards.

## 5. Test Platform

In this section, we describe the V2X testbed devices and configurations used in the implementation and experiment scenarios.

### 5.1. Overview of the Test Platform

**Figure 9:**
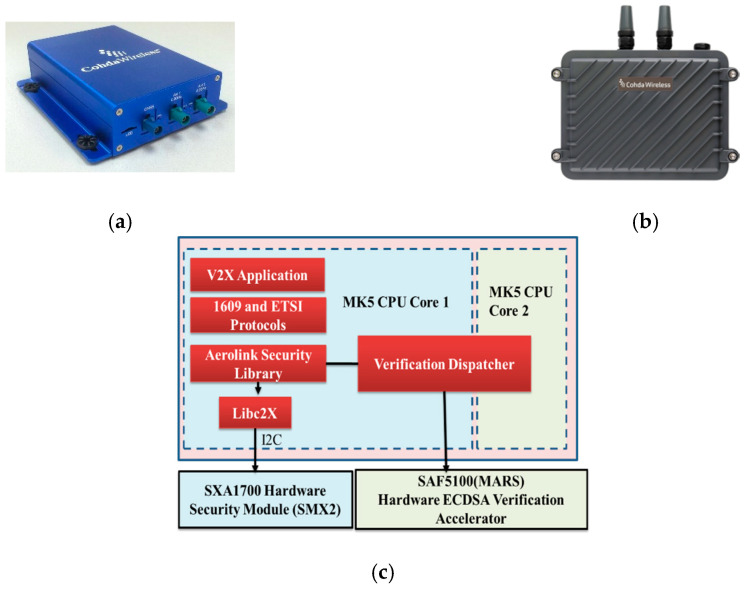
(**a**) Cohda wireless MK5 On-board Unit (OBU), (**b**) Cohda wireless MK5 Road Side Unit (RSU), (**c**) Hardware architecture of MK5 device.

In the experiments, we used commercial DSRC devices, namely, Cohda wireless MK5 devices that are designed to support an integrated platform for the V2X standard protocols and applications. The MK5 devices are provided in two types, MK5-OBU and MK5-RSU. On-board units (OBUs) installed on vehicles run the DSRC communication stack and safety applications, whereas roadside units (RSUs) are installed on the road. OBUs and RSUs are equipped with a GPS receiver, and so they can calculate their position. The MK5 devices have a hardware security module (HSM) that accelerates complex security algorithms such as ECDSA and ECC. In the IEEE 1609.2 and ETSI-103-097 standard protocols, the message authentication signs the messages to be verified using the certificate of the transmitter. MK5’s software development kit (SDK) provides a security library that signs the message with ECDSA using HSM called SXA1700 [33]. Similarly, the message authentication can be verified either by a software module or by the hardware accelerator module. MK5 supports the verification using an ECDSA verification accelerator called SAF5100 [34]. Figure 9a,b show Cohda Wireless OBU and RSU units, while Figure 9c shows the architecture of the software and hardware modules of the MK5 device. Table 2 summarizes the specification of the security modules supported by MK5 devices. We test the performance of both the proposed authentication protocol and the standard protocols using MK5. We also compare the performance of the standard protocols conducted over MK5’s software security module and hardware security module.

### 5.2. Configuration of the Test Platform Overview

**Figure 10:**
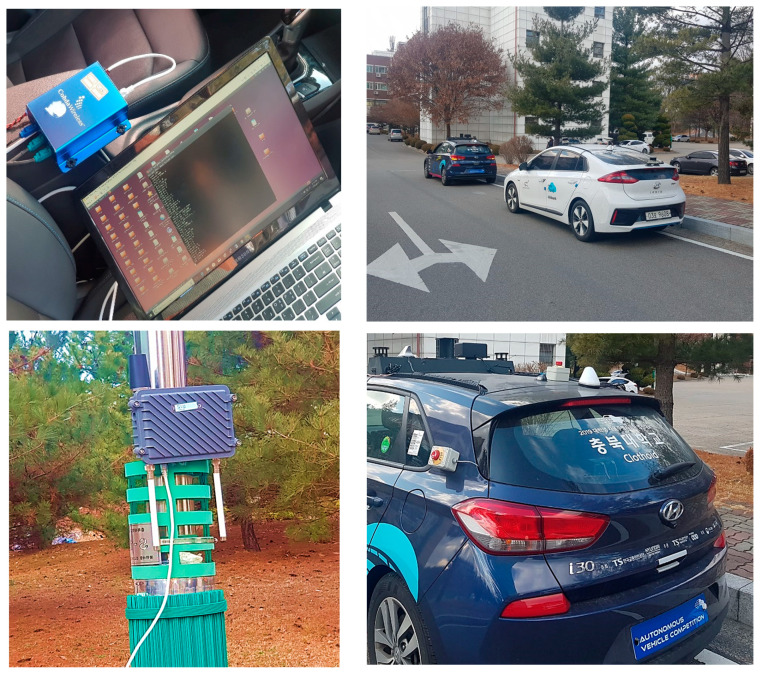
Our test platform consisting of test vehicles, GPS antenna, MK5 OBU devices, and RSU devices.

In our previous work [35], we demonstrated with an outdoor driving test that the speed and mobility of OBU devices have little impact on the V2X wireless performance. In this paper, therefore, we tested the security protocols using our test platform in a stationary condition. The test platform consists of two vehicles equipped with MK5 OBU devices monitored by a portable computer to measure the performance. The GPS antennas are installed on the roof of vehicles, while the RSUs are installed on the road, as shown in Figure 10. During the experiments, we measured the performance of V2X messages using the Wireshark utility [36]. In our test scenarios, OBUs and RSUs are configured to transmit messages with a transmission interval of 100 ms. From an application point of view, there is no difference whether the hardware is an OBU or an RSU. The application configuration defines the behavior of OBU or RSU. ETSI and IEEE 1609 applications are configured with a data rate of 6 Mbps, a TX power of 24 dBm, and a channel ID of 172, 176, and 180—a common setting used for real road. We enabled security using the Aerolink library configuration. The security library utilizes the HSM, SXA1700, to sign messages and utilizes the verification accelerator, SAF5100 ECDSA, to conduct ECDSA verification.

## 6. Measurement of Computation Overhead

In this section, we present the measurement results of the computation overhead of signing and verifying operations of the three protocols: the proposed protocol, IEEE 1609, and 2ETSI-103-097. We configured the MK5 devices to broadcast messages using two modes: with software security module enabled, and with hardware security module enabled. The hardware security module (HSM) cannot be used for the proposed protocol since HSM of MK5 supports only the standard protocols. Thus, the measurements of the proposed protocol are reported using the software security module enabled.

### 6.1. The Proposed Protocol

We implemented the proposed protocol by modifying the IEEE1609 standard. This allows us to measure the computation overhead of signing and verifying various IEEE1609 messages (e.g., BSM, RSA, WSA, and TIM). Each IEEE1609 message is encapsulated with the proposed protocol to support message integrity and identity authentication. Each signing time and verifying time is an average time calculated over 10,000 messages with different sizes ranging between (100–500) bytes and a broadcast rate of 10 Hz. The proposed protocol requires two HMAC-SHA256 operations, one at the sender to sign the message and one at the receiver to verify the message. Then the receiver compares the received MAC with the calculated one to accept or reject the message. Table 3 summarizes the average software signing and verifying time of the proposed protocol implemented on IEEE1609 messages. The software-based signing time for the proposed protocol is close to 0.043 ms, while the software-based verifying time is no greater than 0.058 ms. For the proposed protocol (software-based), Table 4 summarizes the average processing speed in terms of the number of transmitting and receiving messages per second. The average processing speed was measured taking into account the buffering time, processing time, and transmission time. Table 4 demonstrates that the proposed protocol can obtain as high as 47619 signatures and 35714 verifications per second for TIM messages.

### 6.2. IEEE 1609.2 Standard

To measure the computation overhead of the message authentication protocol of IEEE 1609.2 standards, we tested the Cohda Wireless MK5 device with the IEEE 1609 protocol stack. We configured the MK5 devices to broadcast BSM, RSA, WSA, and TIM messages using two operation modes: software-based signing, and hardware-based signing. IEEE 1609.2 security standard employs ECDSA NIST P256 with SHA 256 for signing and verifying the messages. We measured the signing time of various IEEE 1609 messages of size ranging between 100–500 bytes.

As we described in Section 4, all messages of IEEE 1609 are either in a full-certificate form or digest-only form. In Table 5, we report verifying time only for the full-certificate messages. A BSM with a full certificate is transmitted approximately every 500 ms (once in every five transmissions),

Table 5 summarizes the average signing time and verifying time of IEEE1609 messages calculated over 10,000 messages using a software module and a hardware module. The software-based verifying time is excessively long (as long as 28.5 ms for BSM). Even with the hardware module, the verifying time is still very long (as long as 6.12 ms). Table 6 summarizes the average processing speed of signatures and verifications per second for IEEE1609 messages. Table 6 shows that the highest processing speed (for TIM message) in the case of the software module is as low as only 54 signatures and 53 verifications per second. Even for the hardware module case, it is still as low as 255 signatures and 282 verifications per second. Such speed is not acceptable for high traffic roads.

### 6.3. ETSI-103-097 Standard

To evaluate the computation overhead of the message authentication protocol based on the ETSI standard, we configured the Cohda wireless MK5 devices by installing the ETSI protocol stack. We configured the MK5 devices to broadcast CAM, MAP, DENM, and IVI messages using two operation modes: software-based signing, and hardware-based signing. ETSI-103-097 security standard employs ECDSA NIST P256 with SHA 256 for signing and verifying the messages. We measured the signing time of various ETSI messages of size ranging between 100–500 bytes. All messages are broadcasted at 10 Hz transmission frequency (100 ms interval), and every message is attached with a signature and a full certificate.

Table 7 shows the average signing and verifying time of ETSI V2X messages using the software security module, hardware security module. Each signing time and verifying time is an average time calculated over 10,000 messages. We can observe that MAP and DENM messages have a longer signing and verifying time due to their larger size than CAM and IVI messages. The software-based verifying time is excessively long (as long as 32.08 ms for MAP). Even with the hardware module, the verifying time is still very long (as long as 7 ms).

Table 8 summarizes the average processing speed of transmitting and receiving messages per second for the ETSI-103-097 standard. Table 8 shows that receiving messages processing speed ranging from 31 to 46 with a software module (148 to 248 with a hardware module), which is 144 times slower than the proposed reported in Table 4.

According to the requirements of standard DSRC communication of V2X [37], each vehicle broadcasts a beacon message (e.g., BSM, CAM) every 100 ms. In high traffic roads, the number of vehicles within a common wireless range of 300 m can be easily as high as 100 vehicles. When each vehicle broadcasts every 100 ms, every vehicle must verify 1000 messages per second.

Table 4, Table 6 and Table 8 demonstrate that only the proposed protocol can satisfy the requirement of verifying 1000 messages per second, as it can verify 25,000 BSM messages per second using a software security module, as shown in Table 4. In contrast, IEEE1609 and ETSI-103-097 standards respectively, obtain a verification speed of only 163 and 183 messages per second even with HSM, while 35 and 36 messages per second with the software module. Consequently, standard protocols based ECDSA cannot satisfy the DSRC requirements in high-density scenarios, whereas the proposed protocol can easily satisfy the requirements even with the low-cost software module.

## 7. Measurement of Communication Performance

In this section, we present the measured communication overhead of the proposed method compared with the standard methods. The first metric is packet delivery latency due to security processing overhead, and the second metric is the packet verification ratio.

### 7.1. Average End-to-End Delay

The average end-to-end authentication delay consists of the processing time, singing time, transmission time, and verifying time. It is calculated using Equation (2) which represents the end-to-end total delay for n transmitted messages over the total number of messages tried:

(2) $$Avg\;Msg\;delay=\frac{\sum_{m=1}^{\;Msgsent_{n}\;}\left(t_{processing}+t_{signing}+t_{trans\;}+t_{verifing}\right)}{num\;of\;messages}$$

In our experiments, we used two vehicles, one configured as a transmitter and the other as a receiver. We measured the end-to-end delays for the three protocols: The proposed protocol, IEEE 1609.2 standard, and ETSI-103-097. The tests are conducted with three configurations: (1) with software security module, (2) with the hardware security module, and (3) with security disabled.

**Figure 11:**
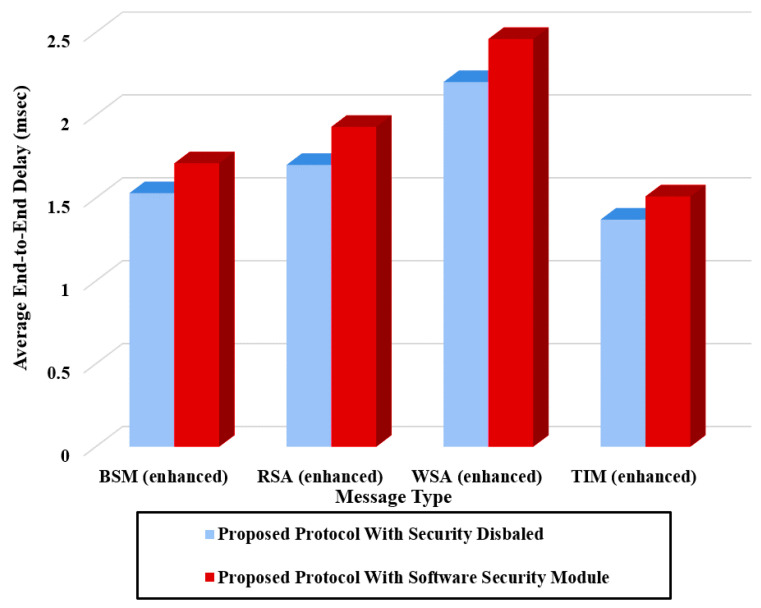
Average end-to-end delay for the proposed protocol applied to IEEE1609.

Figure 11 shows the average end-to-end delay of the proposed protocol implemented on IEEE1609 messages measured with the software security module. From Figure 11, we observe that the proposed protocol substantially reduces the end-to-end delay compared with the standards. It exhibits an end-to-end delay of less than 2.5 ms due to the negligible computation overhead of its HMAC.

**Figure 12:**
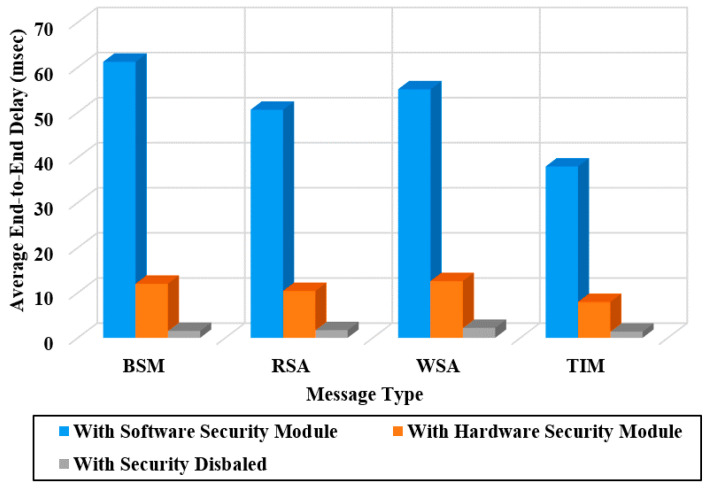
Average end-to-end delay measured for V2X messages of the IEEE 1609 standard.

Figure 12 shows the average end-to-end delay of IEEE1609 messages of four types (BSM, RSA, WSA, and TIM) in a full-certificate form. The end-to-end delay for IEEE1609 messages using the software security module is 60 ms due to the expensive computation of ECDSA signing and verifying operations. Even with the hardware security module, the delay is still very long (as long as 10 ms). It is 24 times longer delay compared with the proposed protocol having a delay of 2.5 ms.

**Figure 13:**
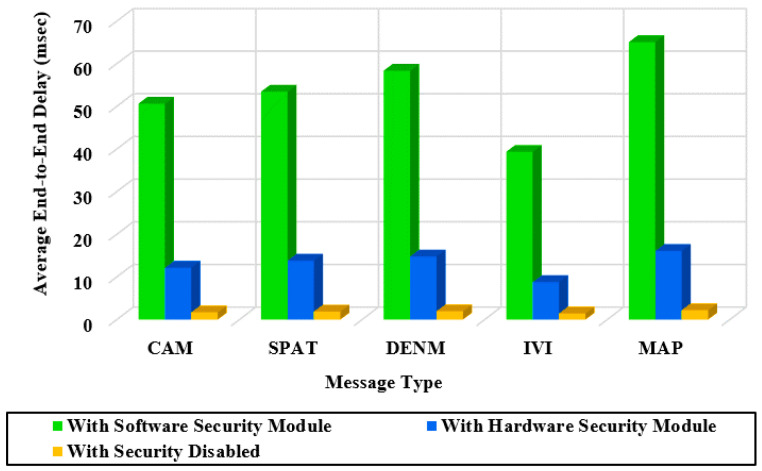
End-to-end delay measured for V2X messages of the ETSI standard.

Figure 13 shows the average end-to-end delay of ETSI-103-097 messages of five types (CAM, SPAT, DENM, IVI, and MAP) in a full-certificate form. The ETSI-103-097 messages exhibit long end-to-end delays up to 65 ms when the software module is used. Even with the hardware module, the end-to-end delay is still very long (as long as 15 ms). Figure 11, Figure 12 and Figure 13 show that when the security is disabled the end-to-end delay for the three protocols exhibits about 2 ms. Note this is close to the delay of the proposed protocol (2.5 ms) with a software module.

**Figure 14:**
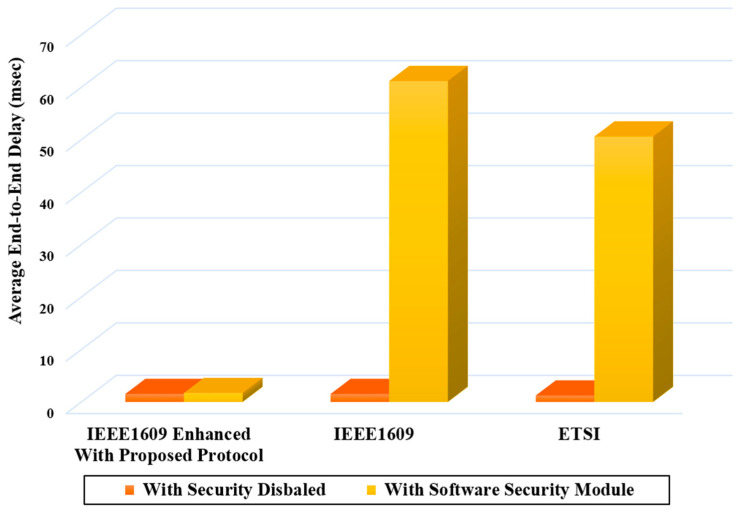
End-to-end delay for IEEE1609 enhanced with proposed protocol, IEEE1609 Standard with software module, and ETSI Standard with software module.

In Figure 14, the average end-to-end delay of IEEE1609 enhanced with the proposed protocol is compared with the case of IEEE1609.2 and ETSI-103-097 standards. From the above experiment of delay measurement, we notice that the proposed protocol only using a low-cost software security module provides a significant reduction in the end-to-end delay compared with the two standard protocols even with a hardware module.

### 7.2. Packet Verification Ratio

The packet verification ratio represents the receiver’s ability of the verification process of the received packets. It is measured by counting the number of received packets that are finished with the verification process without exceeding the capacity of the verification module. The packet verification ratio is defined by Equation (3):

(3) $$\mathrm{Verification}\;\mathrm{Ratio}\;=\;\frac{\mathrm{The}\;\mathrm{number}\;\mathrm{of}\;\mathrm{verified}\;\mathrm{packets}\;}{\mathrm{The}\;\mathrm{number}\;\mathrm{of}\;\mathrm{transmitted}\;\mathrm{packets}}$$

For all three protocols (the proposed, IEEE 1609.2, ETSI-103-097) measured in our experiments, the verification process is conducted by the security layer of the receiver. If the verification is completed, the corresponding counter is incremented to calculate the verification ratio of Equation (3). Since both IEEE1609.2 and ETSI-103-097 standards employ ECDSA for message signature verification, we compare the packet verification ratio of the proposed protocol with only IEEE 1609.2. In both protocols, the messages are queued in the receive buffer with an expiration time of 100 ms until the verification module is freed up. When the verification process exceeds the limit, the packet pending in the FIFO is terminated from the FIFO, since the verification module cannot catch up with the reception rate. In this experiment, we assume that there is no malicious modification in the received packets. Therefore, the counter of the verified packets indicates the number of packets that are finished with the verification module and passed to the application layer before the expiration time. To measure the impact of the vehicle density of the V2X network on the verification ratio, we changed the transmission interval of the transmitter vehicle to emulate a different number of transmissions by changing the DSRC transmission interval (the default value is 100 ms). In our experiments, the vehicle density is defined by the number of vehicles within the radius of the wireless range. For a vehicle density of 100 vehicles, we changed the transmission interval to 1 ms, so the transmitter vehicle can emulate 100 transmitters by sending 100 messages during 100 ms.

**Figure 15:**
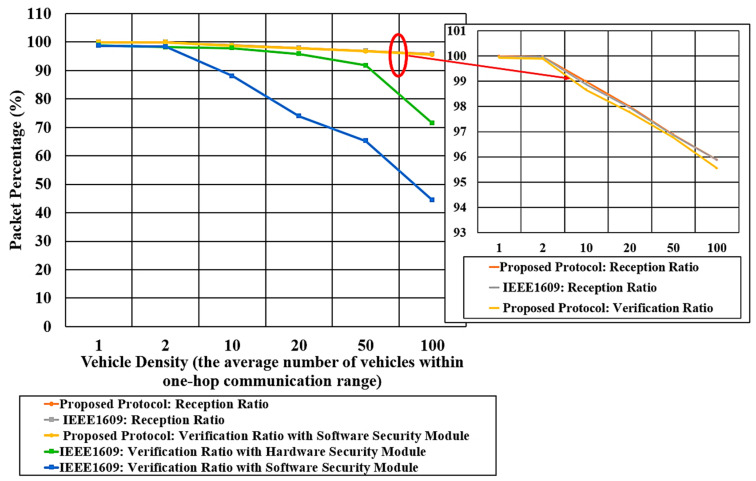
Verification ratio of IEEE1609 and proposed protocol for different vehicle density using software security module.

Figure 15 shows the packet reception ratio for the proposed protocol and IEEE 1609.2 (the upper two graphs) using BSM messages. It also shows the verification ratio for the two protocols using a software security module. In Figure 15, the upper two graphs show that the proposed protocol and IEEE1609.2 have nearly the same packet reception ratio. The reception ratio represents the ratio of packets successfully received at the Physical/MAC layer, which is independent of security protocol. The packet reception ratio is defined by Equation (4):

(4) $$\mathrm{Reception}\;\mathrm{Ratio}\;=\frac{\mathrm{The}\;\mathrm{number}\;\mathrm{of}\;\mathrm{received}\;\mathrm{packets}\;}{\mathrm{The}\;\mathrm{number}\;\mathrm{of}\;\mathrm{transmitted}\;\mathrm{packets}}$$

In Figure 15, the packet reception ratio for the proposed protocol and IEEE1609 is 99% for the vehicle density from 1 to 10, and it decreases to 95% when the vehicle density increases to 100 vehicles within the wireless range. As the vehicle density increases, the packet reception ratio decreases primarily due to the growing chance of packet collisions and bit errors.

According to Table 3, the verification time of the proposed protocol is 40 μs for one BSM message, which is much shorter than the transmission interval of 1 ms. Therefore, we can expect all received messages can be verified. We notice, however, that the verification ratio and reception ratio graphs for the proposed protocol are slightly different. This can be explained by the fact that they have been measured with different runs of experiments that are affected by different multi-tasking scheduling of MK5’s operating system.

Figure 15 also shows that for the vehicle density of 100, the proposed protocol can verify as high as 94% of the packets with the only software module. In contrast, IEEE1609.2 can verify only 46% and 71%, respectively, for the case of using software and hardware module. As discussed in Section 4, IEEE 1609 transmits full-certificate messages once every five transmissions, while it transmits the other message with the only digest. Thus, the verification ratio of IEEE 1609 (with software or hardware module) indicates the average verification ratio of the full-certificate messages and digest-only messages. If IEEE 1609 is configured to transmit only the full-certificate messages, its verification ratio would be even lower. The high verification ratio of the proposed protocol is due to the fact that the verification processing time of the HMAC signature is significantly lower than the ECDSA signature verification as described in Section 6. Therefore, only the proposed protocol can satisfy the requirement of DSRC (verifying 1000 messages per second), and it also can eliminate the needs for expensive hardware security module.

## 8. Performance Comparison with Non-Standard Protocols

In this section, we present the measured communication overhead and computation overhead of the proposed method compared with the non-standard methods 2FLIP [12] and TESLA [14].

### 8.1. Communication Overhead

We first compare the proposed protocol with the non-standard security protocols 2FLIP [12] and TESLA [14]. 2FLIP supports message authentication using HMAC and biometric authentication. Its message structure is represented by Equation (5):

(5) $$PID_{i,ts}\left|\left|\sigma_{i,ts}\right|\right|T_{s}||m$$

Refer to [12] for details. The overhead of 2FLIP comprises $PID_{i,ts}$ of 23 bytes, $\sigma_{i,ts}$ of 20 bytes, and timestamp of 4 bytes leading to a total overhead of 47 bytes.

In [14], the TESLA protocol supports message authentication using the MAC protocol and the one-way hash function. TESLA employs a delayed key disclosure by piggybacking the keys in the next message to generate the MAC for the current message. The message structure of TESLA is illustrated in Equation (6):

(6) $$Cert.Digest||MAC_{ki}(m_{i,j}\left|\left|TS\right)\right|\left|m_{i,j}\left|\left|Ts\right|\right|k_{i-1}\right||sig\left(m_{i,j},k_{i-1},Ts\right)$$

Refer to [14,26] for details. The overhead of TESLA is:

(7) $$\begin{array}{c} \left|Cert.Digest\right|+\left|MAC_{ki}\right|+\left|Ts\right|+\left|k_{i-1}\right|+\left|sig\right||=8+32+6+32+64\\ \;=\;142\;\mathrm{bytes} \end{array}$$

Table 9 compares the communication overhead of the proposed protocol against 2FLIP and TESLA. The proposed protocol incurs a 20% smaller overhead than 2FLIP, and four times smaller overhead than TESLA.

### 8.2. Computation Overhead

We then compare the computation overhead of the proposed method with the non-standard methods 2FLIP [12] and TESLA [14]. We implemented 2FLIP, and TESLA by modifying the IEEE1609 standard. This allows us to measure the computation overhead of signing and verifying various IEEE1609 messages (e.g., BSM, RSA, WSA, and TIM). Each IEEE1609 message is encapsulated with the 2FLIP and TESLA protocols to support message authentication. The signature generation time of 2FLIP comprises one MAC calculation and seven hash operations (7Th + TMAC), while the verifying time is (2Th + TMAC) as mentioned in [12].

The signature generation time of TESLA comprises one ECDSA signing time, one MAC time, and hashing time. The verification time comprises one ECDSA verifying time, one MAC time, key disclosure delay interval, and hash time. The average key disclosure interval during experiments is 13 ms, which defines the time the receiver should wait for the sender to disclose the authentication key to verify the message. We configured the V2X OBU devices to broadcast messages using a software security module enabled for a fair comparison.

The average signing and verifying time of 2FLIP, TESLA, and proposed protocol are shown in Table 10. For the case of BSM, the verification time is 0.13ms and 42.3ms respectively for 2FLIP and TESLA, while the proposed method gives a substantially shorter verification time of 0.04ms.

**Figure 16:**
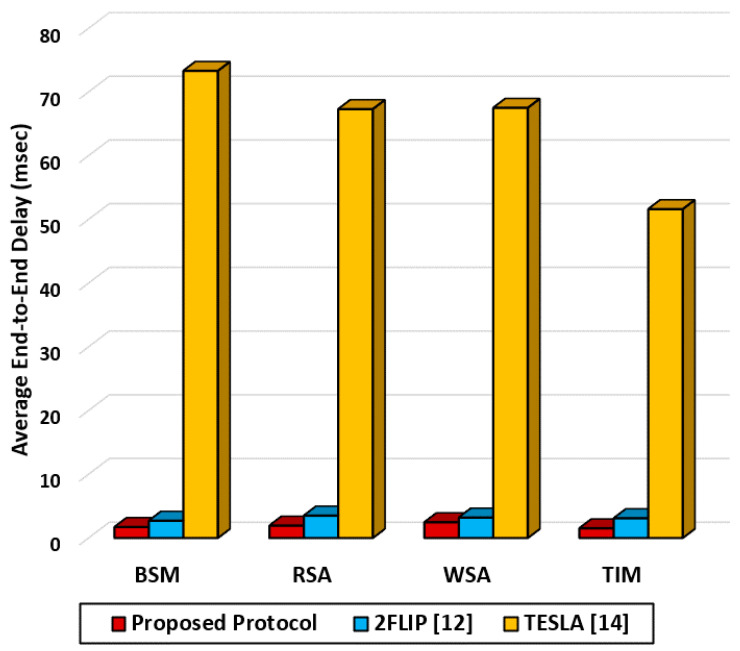
Average end-to-end delay for the proposed protocol and non-standard security protocols using IEEE1609 different messages.

Table 11 compares the processing speed of the three protocols. In the BSM case, the verification speed for 2FLIP and TESLA is 7692 and 23 messages per second, while the proposed protocol provides a significantly faster verification speed of 25,000 messages per second. Figure 16 shows the average End-to-End delay of the three protocols. The proposed protocol reduces the end-to-end delay by 28 times compared with TESLA, while it reduces the delay by 14% compared with 2FLIP. The TESLA protocol exhibits long end-to-end delay up to 72 ms due to its long verification time.

## 9. Conclusions

In this paper, we presented the performance of the proposed light-weight security protocol for V2X communications using a real network with commercial V2X devices. We compared the communication and computation cost of the proposed protocol based on the hash chain with the IEEE 1609 and ETSI-103-097 standards based on ECDSA. We also compared the cost of the proposed protocol with non-standard security protocols (2FLIP and TESLA) based on HMAC. The proposed protocol achieves substantial speed-up in authentication processing with privacy preservation by using a short MAC signature and a pre-generated pseudo-identity.

In contrast to the proposed protocol, the standards employ an ECDSA signature for each message, which imposes excessively high computation overhead. We found that the proposed protocol incurs an overhead of only 40 bytes for OBU messages, which is up to a 6-fold reduction compared with standards and a 4-fold reduction compared with non-standard TESLA protocol. Using extensive experiments with real V2X networks, we demonstrated that the proposed protocol can provide a verification speed as high as 25,000 BSM messages per second using only a low-cost software security module. However, the IEEE1609.2 and ETSI-103-097 standards, respectively, obtain a verification speed of only 163 and 183 messages with a full certificate per second even with a hardware security module. Their verification speed drops further to 35 and 36 messages per second when the software module is used. As for the non-standard protocols, while 2FLIP can verify up to 19230 messages, TESLA can verify only 54 messages per second. Only the proposed protocol and 2FLIP protocol can satisfy the verification-speed requirement of DSRC: verifying 1000 messages per second. The proposed protocol can significantly reduce the computation and communication overhead of message authentication, and thus overcome the serious limitation of the current V2X standards. It is especially effective for time-critical message authentication for V2X networks in high-density scenarios. To the best of our knowledge, no previous work reported the extensive performance measurement of various V2X security protocols in a real network.

## Figures and Tables

**Table 1:** Communication overhead comparison between the proposed protocol and two standards.

	Total Security Overhead Size (Bytes)	Signing and Verifying Algorithm
Proposed Protocol	In the case of OBU: 40	HMAC based on a pre-shared hash chain
	In the case of RSU: 201	
ETSI -103-097	In the case of OBU with full certificate:234	ECDSA nist P256 with sha 256
	In the case of OBU with certificate Digest:90	
	In the case of RSU with full certificate: (234–267)	
IEEE 1609.2	In the case of OBU with full certificate:180	ECDSA nist P256
	In the case of OBU with certificate Digest:93	
	In the case of RSU with full certificate:(224–232)	

**Table 2:** Hardware and software specification of Cohda wireless MK5 devices.

Parameters	Description
Security standards version	IEEE1609.2-2016 v3, ETSI 103097 v1.2.5 2016-05
MK5 firmware version	Release-15 stack software
Cryptographic library	Embedded Aerolink library
Hardware security module	NXP-SXA1700 hardware security module
ECDSA verification module	SAF5100 ECDSA verification accelerator

**Table 3:** Signing and verifying time of the proposed protocol implemented on IEEE1609 messages.

Average Time (ms)	BSM	RSA	WSA	TIM
Software signing	0.036	0.024	0.043	0.021
Software verifying	0.040	0.033	0.058	0.028

**Table 4:** Comparison of Processing Speed For transmitting and receiving messages of the proposed protocol (Software-Based Implementation).

Message Type	BSM	RSA	WSA	TIM
Average number of signed messages per second	27,776	41,665	23,255	47,619
Average number of verified messages per second	25,000	30,303	17,241	35,714

**Table 5:** IEEE1609 Messages signing and verifying time using software and hardware on mk5 wireless units.

Average Time (ms)	BSM	RSA	WSA	TIM
Software signing	29.3	23.74	26.17	18.41
Hardware signing	5.75	4.11	5.58	3.91
Software verifying	28.5	25.64	26.95	18.72
Hardware verifying	6.12	5.78	6.01	3.54

**Table 6:** Comparison of Processing Speed For transmitting and receiving messages of the IEEE1609 in case of software and hardware.

Message Type	BSM	RSA	WSA	TIM
Average number of signed messages per second using software module	31	42	38	54
Average number of signed messages per second using hardware module	173	243	179	255
Average number of verified messages per second using software module	35	39	37	53
Average number of verified messages per second using hardware module	163	173	166	282

**Table 7:** ETSI Messages signing and verifying time using software and hardware on mk5 wireless units.

Average Time (ms)	CAM	SPAT	DENM	IVI	MAP
Software signing	22.11	25.67	27.43	17.11	29.98
Hardware signing	5.31	6.10	7.18	3.87	7.37
Software verifying	27.11	25.66	29.12	21.32	32.08
Hardware verifying	5.45	5.61	5.81	4.02	6.73

**Table 8:** Comparison of processing speed for transmitting and receiving messages of the ETSI standard in case of software and hardware.

Message Type	CAM	SPAT	DENM	IVI	MAP
Average number of signed messages per second using software module	45	38	36	58	32
Average number of signed messages per second using hardware module	188	163	139	258	135
Average number of verified messages per second using software module	36	38	34	46	31
Average number of verified messages per second using hardware module	183	178	172	248	148

**Table 9:** Communication overhead comparison Between the proposed protocol and non-standard protocols for V2V messages.

Protocol	Total Security Overhead Size (Bytes)	Signing and Verifying Algorithm
2FLIP [12]	47	HMAC and biometric authentication
TESLA [14]	142	HMAC and delayed key disclosure
Proposed Protocol	40	HMAC based on a pre-shared hash chain

**Table 10:** Signing and verifying time of proposed protocol and non-standard security protocols implemented on IEEE1609 messages.

Average Time (ms)	Protocol	BSM	RSA	WSA	TIM
Software signing	2FLIP	0.126	0.084	0.088	0.045
	TESLA	29.3	26.2	26.1	18.4
	Proposed Protocol	0.036	0.024	0.043	0.021
Software verifying	2FLIP	0.13	0.093	0.103	0.052
	TESLA	42.3	39.2	39	31.7
	Proposed Protocol	0.040	0.033	0.058	0.028

**Table 11:** Comparison of processing speed for transmitting and receiving messages of proposed protocol and non-standard protocols (software-based implementation).

Average Message Number	Protocol	BSM	RSA	WSA	TIM
Average number of signed messages per second	2FLIP	7936	11,904	11,363	22,222
	TESLA	34	38	38	54
	Proposed protocol	27,776	41,665	23,255	47,619
Average number of verified messages per second	2FLIP	7692	10,752	9708	19,230
	TESLA	23	25	25	31
	Proposed protocol	25,000	30,303	17,241	35,714
